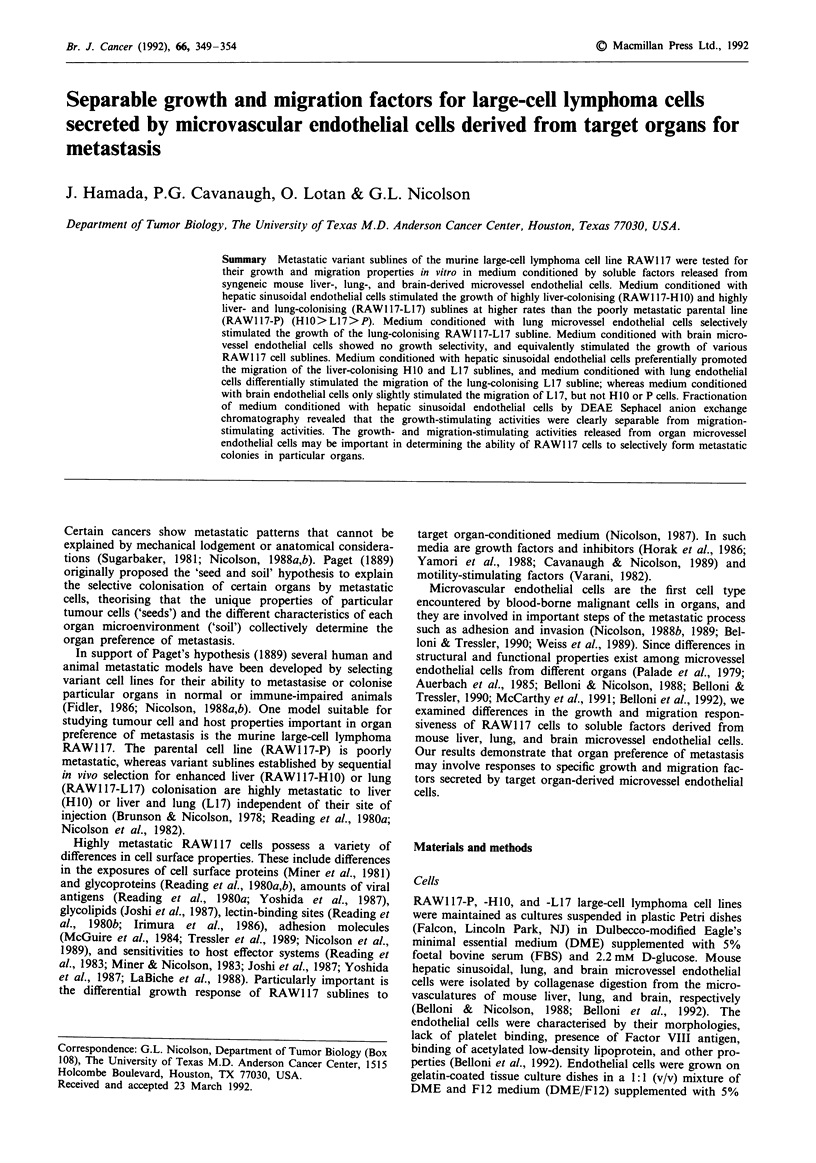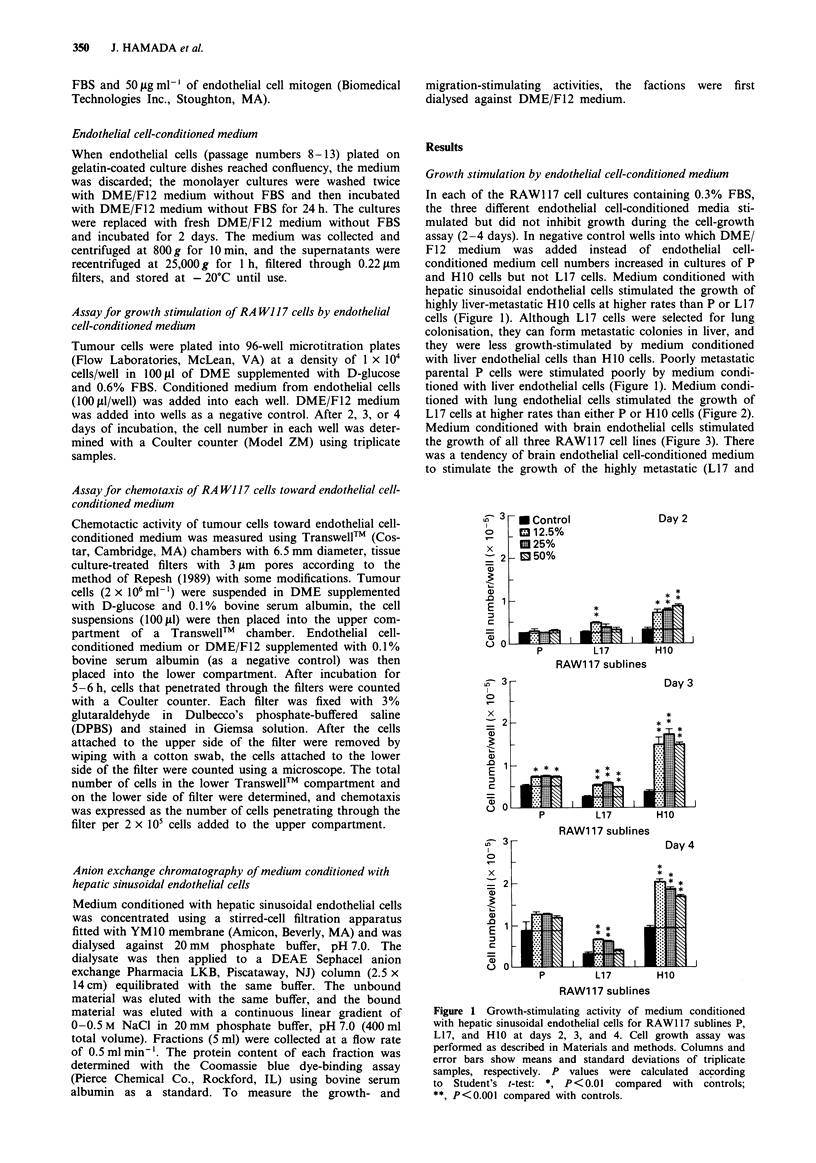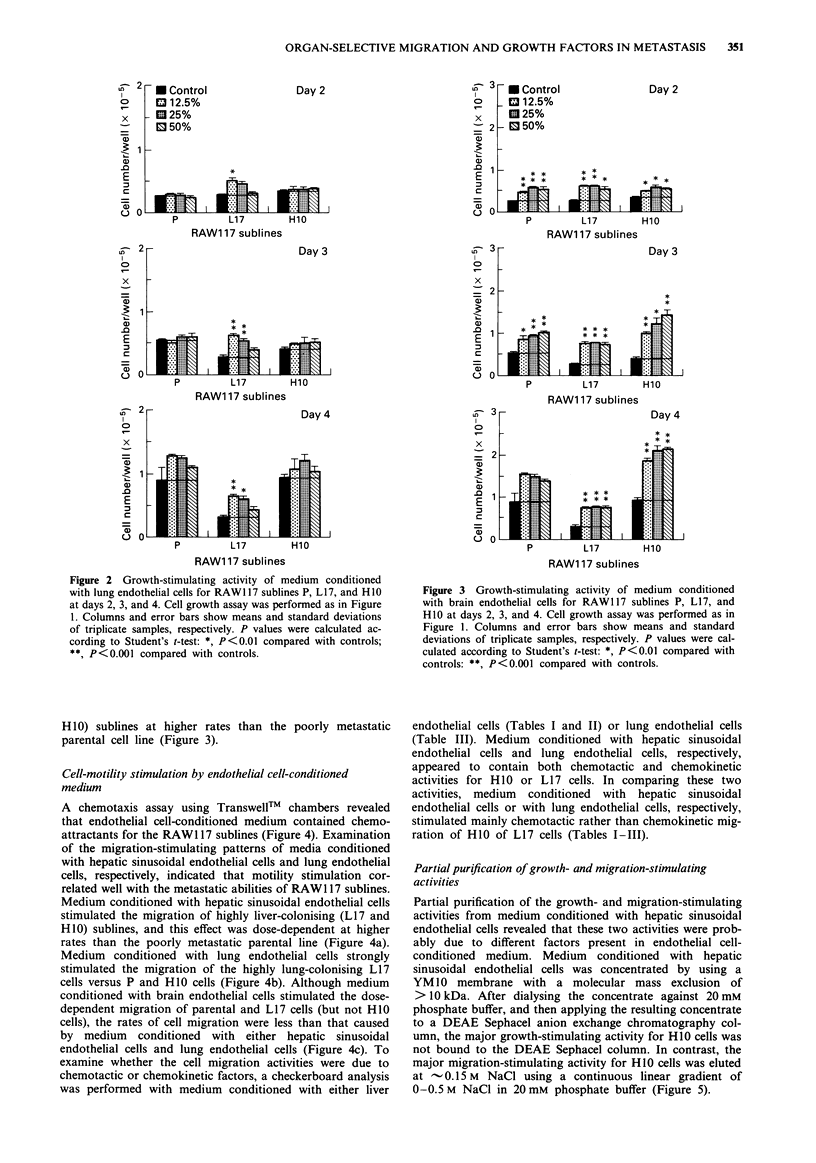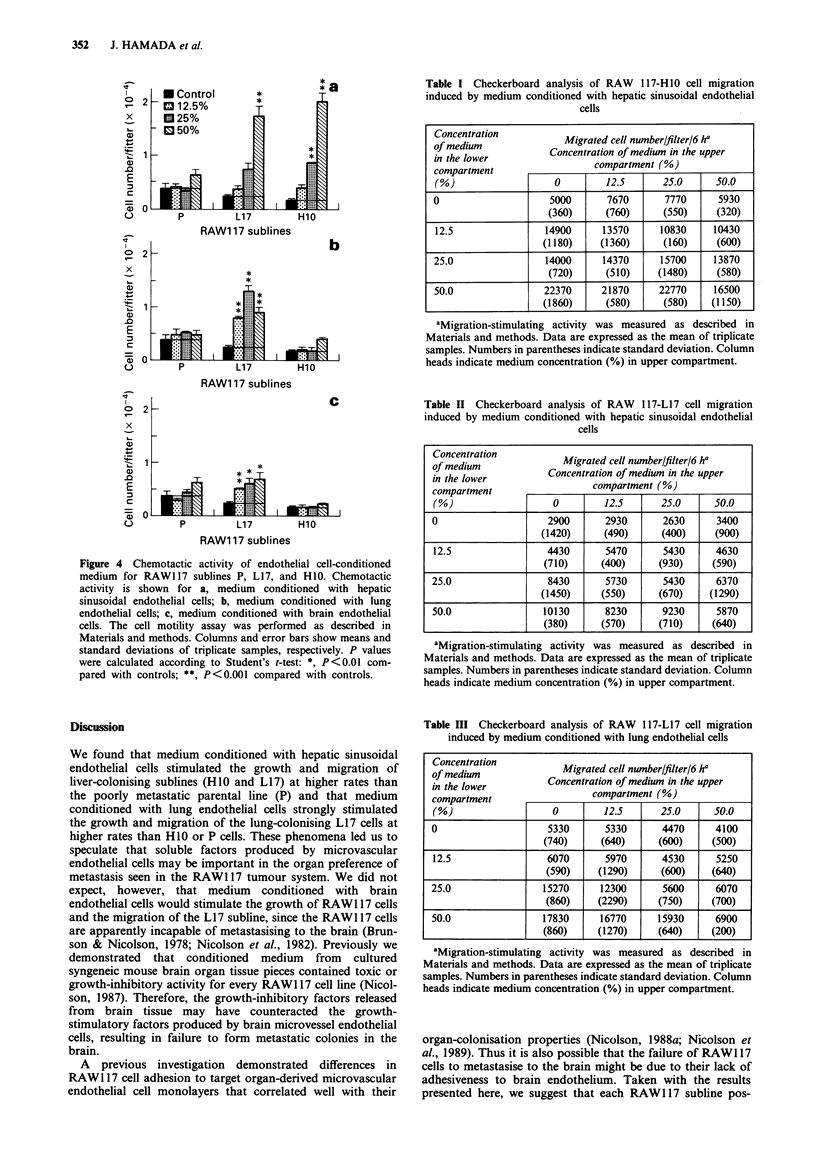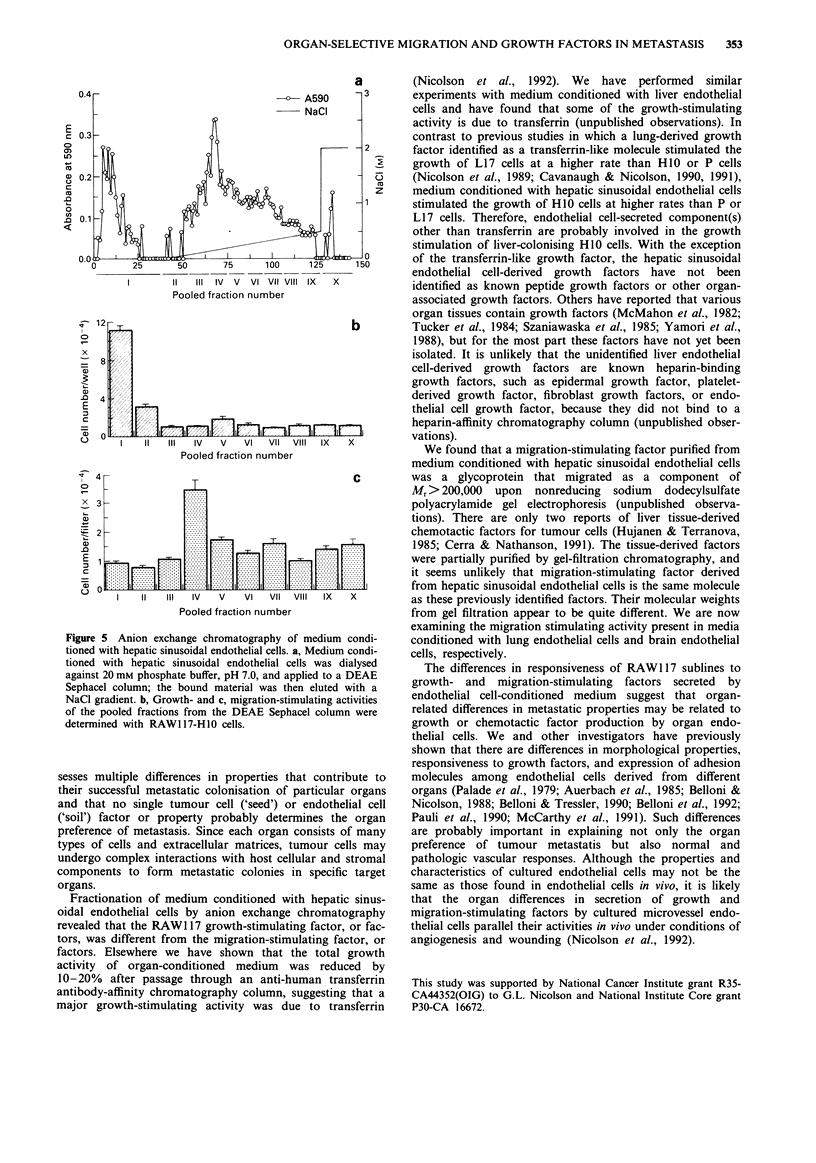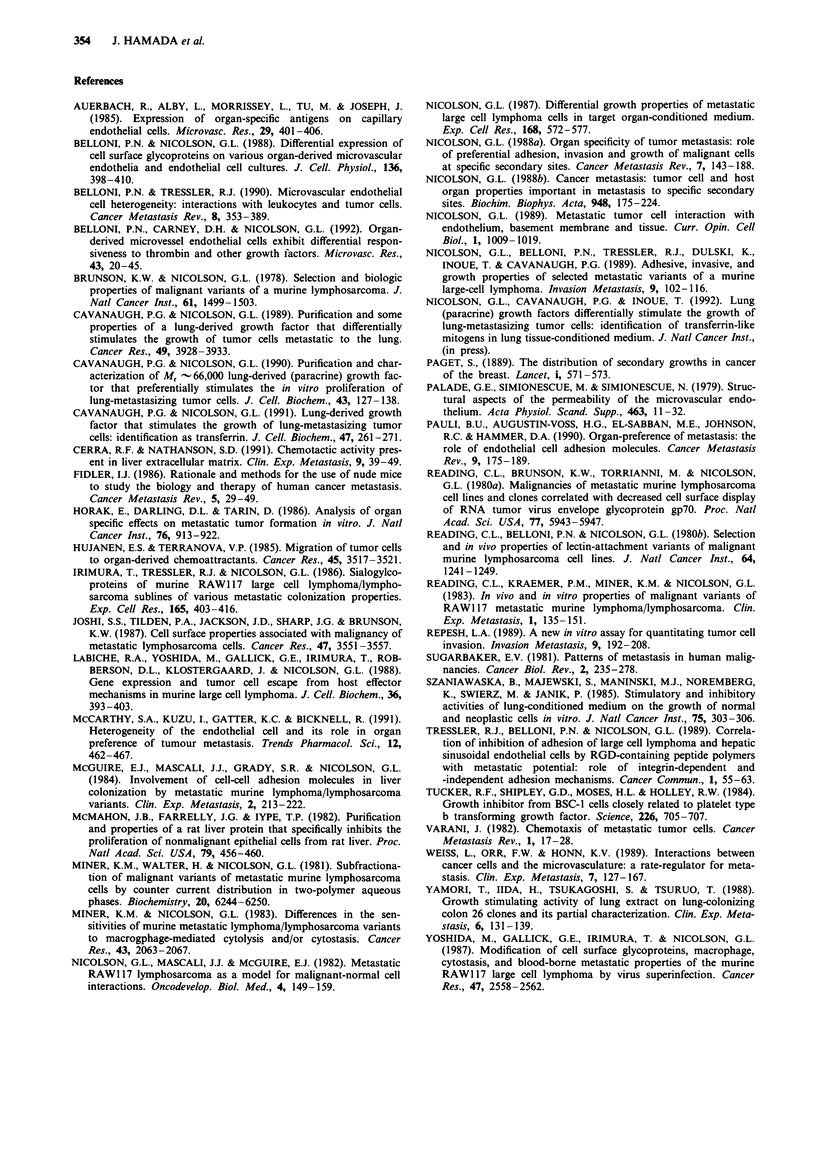# Separable growth and migration factors for large-cell lymphoma cells secreted by microvascular endothelial cells derived from target organs for metastasis.

**DOI:** 10.1038/bjc.1992.269

**Published:** 1992-08

**Authors:** J. Hamada, P. G. Cavanaugh, O. Lotan, G. L. Nicolson

**Affiliations:** Department of Tumor Biology, University of Texas M.D. Anderson Cancer Center, Houston 77030.

## Abstract

Metastatic variant sublines of the murine large-cell lymphoma cell line RAW117 were tested for their growth and migration properties in vitro in medium conditioned by soluble factors released from syngeneic mouse liver-, lung-, and brain-derived microvessel endothelial cells. Medium conditioned with hepatic sinusoidal endothelial cells stimulated the growth of highly liver-colonising (RAW117-H10) and highly liver- and lung-colonising (RAW117-L17) sublines at higher rates than the poorly metastatic parental line (RAW117-P) (H10 greater than L17 greater than P). Medium conditioned with lung microvessel endothelial cells selectively stimulated the growth of the lung-colonising RAW117-L17 subline. Medium conditioned with brain microvessel endothelial cells showed no growth selectivity, and equivalently stimulated the growth of various RAW117 cell sublines. Medium conditioned with hepatic sinusoidal endothelial cells preferentially promoted the migration of the liver-colonising H10 and L17 sublines, and medium conditioned with lung endothelial cells differentially stimulated the migration of the lung-colonising L17 subline; whereas medium conditioned with brain endothelial cells only slightly stimulated the migration of L17, but not H10 or P cells. Fractionation of medium conditioned with hepatic sinusoidal endothelial cells by DEAE Sephacel anion exchange chromatography revealed that the growth-stimulating activities were clearly separable from migration-stimulating activities. The growth- and migration-stimulating activities released from organ microvessel endothelial cells may be important in determining the ability of RAW117 cells to selectively form metastatic colonies in particular organs.


Br. J. Cancer (1992), 66, 349–354 © Macmillan Press Ltd., 1992

### Separable growth and migration factors for large-cell lymphoma cells secreted by microvascular endothelial cells derived from target organs for metastasis

J. Hamada, P.G. Cavanaugh, O. Lotan & G.L. Nicolson

*Department of Tumor Biology, The University of Texas M.D. Anderson Cancer Center, Houston, Texas 77030, USA.*

**Summary** Metastatic variant sublines of the murine large-cell lymphoma cell line RAW117 were tested for their growth and migration properties *in vitro* in medium conditioned by soluble factors released from syngeneic mouse liver-, lung-, and brain-derived microvessel endothelial cells. Medium conditioned with hepatic sinusoidal endothelial cells stimulated the growth of highly liver-colonising (RAW117-H10) and highly liver- and lung-colonising (RAW117-L17) sublines at higher rates than the poorly metastatic parental line (RAW117-P) (H10>L17>*P*). Medium conditioned with lung microvessel endothelial cells selectively stimulated the growth of the lung-colonising RAW117-L17 subline. Medium conditioned with brain microvessel endothelial cells showed no growth selectivity, and equivalently stimulated the growth of various RAW117 cell sublines. Medium conditioned with hepatic sinusoidal endothelial cells preferentially promoted the migration of the liver-colonising H10 and L17 sublines, and medium conditioned with lung endothelial cells differentially stimulated the migration of the lung-colonising L17 subline; whereas medium conditioned with brain endothelial cells only slightly stimulated the migration of L17, but not H10 or P cells. Fractionation of medium conditioned with hepatic sinusoidal endothelial cells by DEAE Sephacel anion exchange chromatography revealed that the growth-stimulating activities were clearly separable from migration-stimulating activities. The growth- and migration-stimulating activities released from organ microvessel endothelial cells may be important in determining the ability of RAW117 cells to selectively form metastatic colonies in particular organs.

Certain cancers show metastatic patterns that cannot be explained by mechanical lodgement or anatomical considerations (Sugarbaker, 1981; Nicolson, 1988*a*,*b*). Paget (1889) originally proposed the 'seed and soil' hypothesis to explain the selective colonisation of certain organs by metastatic cells, theorising that the unique properties of particular tumour cells ('seeds') and the different characteristics of each organ microenvironment ('soil') collectively determine the organ preference of metastasis.

In support of Paget's hypothesis (1889) several human and animal metastatic models have been developed by selecting variant cell lines for their ability to metastasise or colonise particular organs in normal or immune-impaired animals (Fidler, 1986; Nicolson, 1988*a*,*b*). One model suitable for studying tumour cell and host properties important in organ preference of metastasis is the murine large-cell lymphoma RAW117. The parental cell line (RAW117-P) is poorly metastatic, whereas variant sublines established by sequential *in vivo* selection for enhanced liver (RAW117-H10) or lung (RAW117-L17) colonisation are highly metastatic to liver (H10) or liver and lung (L17) independent of their site of injection (Brunson & Nicolson, 1978; Reading *et al.*, 1980*a*; Nicolson *et al.*, 1982).

Highly metastatic RAW117 cells possess a variety of differences in cell surface properties. These include differences in the exposures of cell surface proteins (Miner *et al.*, 1981) and glycoproteins (Reading *et al.*, 1980*a*,*b*), amounts of viral antigens (Reading *et al.*, 1980*a*; Yoshida *et al.*, 1987), glycolipids (Joshi *et al.*, 1987), lectin-binding sites (Reading *et al.*, 1980*b*; Irimura *et al.*, 1986), adhesion molecules (McGuire *et al.*, 1984; Tressler *et al.*, 1989; Nicolson *et al.*, 1989), and sensitivities to host effector systems (Reading *et al.*, 1983; Miner & Nicolson, 1983; Joshi *et al.*, 1987; Yoshida *et al.*, 1987; LaBiche *et al.*, 1988). Particularly important is the differential growth response of RAW117 sublines to target organ-conditioned medium (Nicolson, 1987). In such media are growth factors and inhibitors (Horak *et al.*, 1986; Yamori *et al.*, 1988; Cavanaugh & Nicolson, 1989) and motility-stimulating factors (Varani, 1982).

Microvascular endothelial cells are the first cell type encountered by blood-borne malignant cells in organs, and they are involved in important steps of the metastatic process such as adhesion and invasion (Nicolson, 1988*b*, 1989; Belloni & Tressler, 1990; Weiss *et al.*, 1989). Since differences in structural and functional properties exist among microvessel endothelial cells from different organs (Palade *et al.*, 1979; Auerbach *et al.*, 1985; Belloni & Nicolson, 1988; Belloni & Tressler, 1990; McCarthy *et al.*, 1991; Belloni *et al.*, 1992), we examined differences in the growth and migration responsiveness of RAW117 cells to soluble factors derived from mouse liver, lung, and brain microvessel endothelial cells. Our results demonstrate that organ preference of metastasis may involve responses to specific growth and migration factors secreted by target organ-derived microvessel endothelial cells.

### Materials and methods

### *Cells*

RAW117-P, -H10, and -L17 large-cell lymphoma cell lines were maintained as cultures suspended in plastic Petri dishes (Falcon, Lincoln Park, NJ) in Dulbecco-modified Eagle's minimal essential medium (DME) supplemented with 5% foetal bovine serum (FBS) and 2.2 mM D-glucose. Mouse hepatic sinusoidal, lung, and brain microvessel endothelial cells were isolated by collagenase digestion from the microvasculatures of mouse liver, lung, and brain, respectively (Belloni & Nicolson, 1988; Belloni *et al.*, 1992). The endothelial cells were characterised by their morphologies, lack of platelet binding, presence of Factor VIII antigen, binding of acetylated low-density lipoprotein, and other properties (Belloni *et al.*, 1992). Endothelial cells were grown on gelatin-coated tissue culture dishes in a 1:1 (v/v) mixture of DME and F12 medium (DME/F12) supplemented with 5%

Correspondence: G.L. Nicolson, Department of Tumor Biology (Box 108), The University of Texas M.D. Anderson Cancer Center, 1515 Holcombe Boulevard, Houston, TX 77030, USA.
Received and accepted 23 March 1992.

FBS and 50 μg ml$^{-1}$ of endothelial cell mitogen (Biomedical Technologies Inc., Stoughton, MA).

*Endothelial cell-conditioned medium*

When endothelial cells (passage numbers 8–13) plated on gelatin-coated culture dishes reached confluency, the medium was discarded; the monolayer cultures were washed twice with DME/F12 medium without FBS and then incubated with DME/F12 medium without FBS for 24 h. The cultures were replaced with fresh DME/F12 medium without FBS and incubated for 2 days. The medium was collected and centrifuged at 800 *g* for 10 min, and the supernatants were recentrifuged at 25,000 *g* for 1 h, filtered through 0.22 μm filters, and stored at − 20°C until use.

*Assay for growth stimulation of RAW117 cells by endothelial cell-conditioned medium*

Tumour cells were plated into 96-well microtitration plates (Flow Laboratories, McLean, VA) at a density of $1 \times 10^4$ cells/well in 100 μl of DME supplemented with D-glucose and 0.6% FBS. Conditioned medium from endothelial cells (100 μl/well) was added into each well. DME/F12 medium was added into wells as a negative control. After 2, 3, or 4 days of incubation, the cell number in each well was determined with a Coulter counter (Model ZM) using triplicate samples.

*Assay for chemotaxis of RAW117 cells toward endothelial cell-conditioned medium*

Chemotactic activity of tumour cells toward endothelial cell-conditioned medium was measured using Transwell™ (Costar, Cambridge, MA) chambers with 6.5 mm diameter, tissue culture-treated filters with 3 μm pores according to the method of Repesh (1989) with some modifications. Tumour cells ($2 \times 10^6$ ml$^{-1}$) were suspended in DME supplemented with D-glucose and 0.1% bovine serum albumin, the cell suspensions (100 μl) were then placed into the upper compartment of a Transwell™ chamber. Endothelial cell-conditioned medium or DME/F12 supplemented with 0.1% bovine serum albumin (as a negative control) was then placed into the lower compartment. After incubation for 5–6 h, cells that penetrated through the filters were counted with a Coulter counter. Each filter was fixed with 3% glutaraldehyde in Dulbecco's phosphate-buffered saline (DPBS) and stained in Giemsa solution. After the cells attached to the upper side of the filter were removed by wiping with a cotton swab, the cells attached to the lower side of the filter were counted using a microscope. The total number of cells in the lower Transwell™ compartment and on the lower side of filter were determined, and chemotaxis was expressed as the number of cells penetrating through the filter per $2 \times 10^5$ cells added to the upper compartment.

*Anion exchange chromatography of medium conditioned with hepatic sinusoidal endothelial cells*

Medium conditioned with hepatic sinusoidal endothelial cells was concentrated using a stirred-cell filtration apparatus fitted with YM10 membrane (Amicon, Beverly, MA) and was dialysed against 20 mM phosphate buffer, pH 7.0. The dialysate was then applied to a DEAE Sephacel anion exchange Pharmacia LKB, Piscataway, NJ) column (2.5 × 14 cm) equilibrated with the same buffer. The unbound material was eluted with the same buffer, and the bound material was eluted with a continuous linear gradient of 0–0.5 M NaCl in 20 mM phosphate buffer, pH 7.0 (400 ml total volume). Fractions (5 ml) were collected at a flow rate of 0.5 ml min$^{-1}$. The protein content of each fraction was determined with the Coomassie blue dye-binding assay (Pierce Chemical Co., Rockford, IL) using bovine serum albumin as a standard. To measure the growth- and migration-stimulating activities, the factions were first dialysed against DME/F12 medium.

### Results

*Growth stimulation by endothelial cell-conditioned medium*

In each of the RAW117 cell cultures containing 0.3% FBS, the three different endothelial cell-conditioned media stimulated but did not inhibit growth during the cell-growth assay (2–4 days). In negative control wells into which DME/F12 medium was added instead of endothelial cell-conditioned medium cell numbers increased in cultures of P and H10 cells but not L17 cells. Medium conditioned with hepatic sinusoidal endothelial cells stimulated the growth of highly liver-metastatic H10 cells at higher rates than P or L17 cells (Figure 1). Although L17 cells were selected for lung colonisation, they can form metastatic colonies in liver, and they were less growth-stimulated by medium conditioned with liver endothelial cells than H10 cells. Poorly metastatic parental P cells were stimulated poorly by medium conditioned with liver endothelial cells (Figure 1). Medium conditioned with lung endothelial cells stimulated the growth of L17 cells at higher rates than either P or H10 cells (Figure 2). Medium conditioned with brain endothelial cells stimulated the growth of all three RAW117 cell lines (Figure 3). There was a tendency of brain endothelial cell-conditioned medium to stimulate the growth of the highly metastatic (L17 and

**Figure 1** Growth-stimulating activity of medium conditioned with hepatic sinusoidal endothelial cells for RAW117 sublines P, L17, and H10 at days 2, 3, and 4. Cell growth assay was performed as described in Materials and methods. Columns and error bars show means and standard deviations of triplicate samples, respectively. *P* values were calculated according to Student's *t*-test: *, $P < 0.01$ compared with controls; **, $P < 0.001$ compared with controls.

Figure 2 Growth-stimulating activity of medium conditioned with lung endothelial cells for RAW117 sublines P, L17, and H10 at days 2, 3, and 4. Cell growth assay was performed as in Figure 1. Columns and error bars show means and standard deviations of triplicate samples, respectively. $P$ values were calculated according to Student's $t$-test: *, $P<0.01$ compared with controls; **, $P<0.001$ compared with controls.

Figure 3 Growth-stimulating activity of medium conditioned with brain endothelial cells for RAW117 sublines P, L17, and H10 at days 2, 3, and 4. Cell growth assay was performed as in Figure 1. Columns and error bars show means and standard deviations of triplicate samples, respectively. $P$ values were calculated according to Student's $t$-test: *, $P<0.01$ compared with controls: **, $P<0.001$ compared with controls.

H10) sublines at higher rates than the poorly metastatic parental cell line (Figure 3).

### *Cell-motility stimulation by endothelial cell-conditioned medium*

A chemotaxis assay using Transwell™ chambers revealed that endothelial cell-conditioned medium contained chemoattractants for the RAW117 sublines (Figure 4). Examination of the migration-stimulating patterns of media conditioned with hepatic sinusoidal endothelial cells and lung endothelial cells, respectively, indicated that motility stimulation correlated well with the metastatic abilities of RAW117 sublines. Medium conditioned with hepatic sinusoidal endothelial cells stimulated the migration of highly liver-colonising (L17 and H10) sublines, and this effect was dose-dependent at higher rates than the poorly metastatic parental line (Figure 4a). Medium conditioned with lung endothelial cells strongly stimulated the migration of the highly lung-colonising L17 cells versus P and H10 cells (Figure 4b). Although medium conditioned with brain endothelial cells stimulated the dose-dependent migration of parental and L17 cells (but not H10 cells), the rates of cell migration were less than that caused by medium conditioned with either hepatic sinusoidal endothelial cells and lung endothelial cells (Figure 4c). To examine whether the cell migration activities were due to chemotactic or chemokinetic factors, a checkerboard analysis was performed with medium conditioned with either liver endothelial cells (Tables I and II) or lung endothelial cells (Table III). Medium conditioned with hepatic sinusoidal endothelial cells and lung endothelial cells, respectively, appeared to contain both chemotactic and chemokinetic activities for H10 or L17 cells. In comparing these two activities, medium conditioned with hepatic sinusoidal endothelial cells or with lung endothelial cells, respectively, stimulated mainly chemotactic rather than chemokinetic migration of H10 of L17 cells (Tables I–III).

### *Partial purification of growth- and migration-stimulating activities*

Partial purification of the growth- and migration-stimulating activities from medium conditioned with hepatic sinusoidal endothelial cells revealed that these two activities were probably due to different factors present in endothelial cell-conditioned medium. Medium conditioned with hepatic sinusoidal endothelial cells was concentrated by using a YM10 membrane with a molecular mass exclusion of >10 kDa. After dialysing the concentrate against 20 mM phosphate buffer, and then applying the resulting concentrate to a DEAE Sephacel anion exchange chromatography column, the major growth-stimulating activity for H10 cells was not bound to the DEAE Sephacel column. In contrast, the major migration-stimulating activity for H10 cells was eluted at ~0.15 M NaCl using a continuous linear gradient of 0–0.5 M NaCl in 20 mM phosphate buffer (Figure 5).

**Figure 4** Chemotactic activity of endothelial cell-conditioned medium for RAW117 sublines P, L17, and H10. Chemotactic activity is shown for **a**, medium conditioned with hepatic sinusoidal endothelial cells; **b**, medium conditioned with lung endothelial cells; **c**, medium conditioned with brain endothelial cells. The cell motility assay was performed as described in Materials and methods. Columns and error bars show means and standard deviations of triplicate samples, respectively. *P* values were calculated according to Student's *t*-test: *, $P<0.01$ compared with controls; **, $P<0.001$ compared with controls.

### Discussion

We found that medium conditioned with hepatic sinusoidal endothelial cells stimulated the growth and migration of liver-colonising sublines (H10 and L17) at higher rates than the poorly metastatic parental line (P) and that medium conditioned with lung endothelial cells strongly stimulated the growth and migration of the lung-colonising L17 cells at higher rates than H10 or P cells. These phenomena led us to speculate that soluble factors produced by microvascular endothelial cells may be important in the organ preference of metastasis seen in the RAW117 tumour system. We did not expect, however, that medium conditioned with brain endothelial cells would stimulate the growth of RAW117 cells and the migration of the L17 subline, since the RAW117 cells are apparently incapable of metastasising to the brain (Brunson & Nicolson, 1978; Nicolson *et al.*, 1982). Previously we demonstrated that conditioned medium from cultured syngeneic mouse brain organ tissue pieces contained toxic or growth-inhibitory activity for every RAW117 cell line (Nicolson, 1987). Therefore, the growth-inhibitory factors released from brain tissue may have counteracted the growth-stimulatory factors produced by brain microvessel endothelial cells, resulting in failure to form metastatic colonies in the brain.

A previous investigation demonstrated differences in RAW117 cell adhesion to target organ-derived microvascular endothelial cell monolayers that correlated well with their organ-colonisation properties (Nicolson, 1988*a*; Nicolson *et al.*, 1989). Thus it is also possible that the failure of RAW117 cells to metastasise to the brain might be due to their lack of adhesiveness to brain endothelium. Taken with the results presented here, we suggest that each RAW117 subline pos-

**Table I** Checkerboard analysis of RAW 117-H10 cell migration induced by medium conditioned with hepatic sinusoidal endothelial cells

| *Concentration of medium in the lower compartment (%)* | *Migrated cell number/filter/6 h*[a] *Concentration of medium in the upper compartment (%)* 0 | 12.5 | 25.0 | 50.0 |
|---|---|---|---|---|
| 0 | 5000 (360) | 7670 (760) | 7770 (550) | 5930 (320) |
| 12.5 | 14900 (1180) | 13570 (1360) | 10830 (160) | 10430 (600) |
| 25.0 | 14000 (720) | 14370 (510) | 15700 (1480) | 13870 (580) |
| 50.0 | 22370 (1860) | 21870 (580) | 22770 (580) | 16500 (1150) |

[a]Migration-stimulating activity was measured as described in Materials and methods. Data are expressed as the mean of triplicate samples. Numbers in parentheses indicate standard deviation. Column heads indicate medium concentration (%) in upper compartment.

**Table II** Checkerboard analysis of RAW 117-L17 cell migration induced by medium conditioned with hepatic sinusoidal endothelial cells

| *Concentration of medium in the lower compartment (%)* | *Migrated cell number/filter/6 h*[a] *Concentration of medium in the upper compartment (%)* 0 | 12.5 | 25.0 | 50.0 |
|---|---|---|---|---|
| 0 | 2900 (1420) | 2930 (490) | 2630 (400) | 3400 (900) |
| 12.5 | 4430 (710) | 5470 (400) | 5430 (930) | 4630 (590) |
| 25.0 | 8430 (1450) | 5730 (550) | 5430 (670) | 6370 (1290) |
| 50.0 | 10130 (380) | 8230 (570) | 9230 (710) | 5870 (640) |

[a]Migration-stimulating activity was measured as described in Materials and methods. Data are expressed as the mean of triplicate samples. Numbers in parentheses indicate standard deviation. Column heads indicate medium concentration (%) in upper compartment.

**Table III** Checkerboard analysis of RAW 117-L17 cell migration induced by medium conditioned with lung endothelial cells

| *Concentration of medium in the lower compartment (%)* | *Migrated cell number/filter/6 h*[a] *Concentration of medium in the upper compartment (%)* 0 | 12.5 | 25.0 | 50.0 |
|---|---|---|---|---|
| 0 | 5330 (740) | 5330 (640) | 4470 (600) | 4100 (500) |
| 12.5 | 6070 (590) | 5970 (1290) | 4530 (600) | 5250 (640) |
| 25.0 | 15270 (860) | 12300 (2290) | 5600 (750) | 6070 (700) |
| 50.0 | 17830 (860) | 16770 (1270) | 15930 (640) | 6900 (200) |

[a]Migration-stimulating activity was measured as described in Materials and methods. Data are expressed as the mean of triplicate samples. Numbers in parentheses indicate standard deviation. Column heads indicate medium concentration (%) in upper compartment.

**Figure 5** Anion exchange chromatography of medium conditioned with hepatic sinusoidal endothelial cells. **a**, Medium conditioned with hepatic sinusoidal endothelial cells was dialysed against 20 mM phosphate buffer, pH 7.0, and applied to a DEAE Sephacel column; the bound material was then eluted with a NaCl gradient. **b**, Growth- and **c**, migration-stimulating activities of the pooled fractions from the DEAE Sephacel column were determined with RAW117-H10 cells.

sesses multiple differences in properties that contribute to their successful metastatic colonisation of particular organs and that no single tumour cell ('seed') or endothelial cell ('soil') factor or property probably determines the organ preference of metastasis. Since each organ consists of many types of cells and extracellular matrices, tumour cells may undergo complex interactions with host cellular and stromal components to form metastatic colonies in specific target organs.

Fractionation of medium conditioned with hepatic sinusoidal endothelial cells by anion exchange chromatography revealed that the RAW117 growth-stimulating factor, or factors, was different from the migration-stimulating factor, or factors. Elsewhere we have shown that the total growth activity of organ-conditioned medium was reduced by 10–20% after passage through an anti-human transferrin antibody-affinity chromatography column, suggesting that a major growth-stimulating activity was due to transferrin (Nicolson *et al.*, 1992). We have performed similar experiments with medium conditioned with liver endothelial cells and have found that some of the growth-stimulating activity is due to transferrin (unpublished observations). In contrast to previous studies in which a lung-derived growth factor identified as a transferrin-like molecule stimulated the growth of L17 cells at a higher rate than H10 or P cells (Nicolson *et al.*, 1989; Cavanaugh & Nicolson, 1990, 1991), medium conditioned with hepatic sinusoidal endothelial cells stimulated the growth of H10 cells at higher rates than P or L17 cells. Therefore, endothelial cell-secreted component(s) other than transferrin are probably involved in the growth stimulation of liver-colonising H10 cells. With the exception of the transferrin-like growth factor, the hepatic sinusoidal endothelial cell-derived growth factors have not been identified as known peptide growth factors or other organ-associated growth factors. Others have reported that various organ tissues contain growth factors (McMahon *et al.*, 1982; Tucker *et al.*, 1984; Szaniawaska *et al.*, 1985; Yamori *et al.*, 1988), but for the most part these factors have not yet been isolated. It is unlikely that the unidentified liver endothelial cell-derived growth factors are known heparin-binding growth factors, such as epidermal growth factor, platelet-derived growth factor, fibroblast growth factors, or endothelial cell growth factor, because they did not bind to a heparin-affinity chromatography column (unpublished observations).

We found that a migration-stimulating factor purified from medium conditioned with hepatic sinusoidal endothelial cells was a glycoprotein that migrated as a component of $M_r > 200,000$ upon nonreducing sodium dodecylsulfate polyacrylamide gel electrophoresis (unpublished observations). There are only two reports of liver tissue-derived chemotactic factors for tumour cells (Hujanen & Terranova, 1985; Cerra & Nathanson, 1991). The tissue-derived factors were partially purified by gel-filtration chromatography, and it seems unlikely that migration-stimulating factor derived from hepatic sinusoidal endothelial cells is the same molecule as these previously identified factors. Their molecular weights from gel filtration appear to be quite different. We are now examining the migration stimulating activity present in media conditioned with lung endothelial cells and brain endothelial cells, respectively.

The differences in responsiveness of RAW117 sublines to growth- and migration-stimulating factors secreted by endothelial cell-conditioned medium suggest that organ-related differences in metastatic properties may be related to growth or chemotactic factor production by organ endothelial cells. We and other investigators have previously shown that there are differences in morphological properties, responsiveness to growth factors, and expression of adhesion molecules among endothelial cells derived from different organs (Palade *et al.*, 1979; Auerbach *et al.*, 1985; Belloni & Nicolson, 1988; Belloni & Tressler, 1990; Belloni *et al.*, 1992; Pauli *et al.*, 1990; McCarthy *et al.*, 1991). Such differences are probably important in explaining not only the organ preference of tumour metastatis but also normal and pathologic vascular responses. Although the properties and characteristics of cultured endothelial cells may not be the same as those found in endothelial cells *in vivo*, it is likely that the organ differences in secretion of growth and migration-stimulating factors by cultured microvessel endothelial cells parallel their activities *in vivo* under conditions of angiogenesis and wounding (Nicolson *et al.*, 1992).

This study was supported by National Cancer Institute grant R35-CA44352(OIG) to G.L. Nicolson and National Institute Core grant P30-CA 16672.

### References

AUERBACH, R., ALBY, L., MORRISSEY, L., TU, M. & JOSEPH, J. (1985). Expression of organ-specific antigens on capillary endothelial cells. *Microvasc. Res.*, **29**, 401–406.

BELLONI, P.N. & NICOLSON, G.L. (1988). Differential expression of cell surface glycoproteins on various organ-derived microvascular endothelia and endothelial cell cultures. *J. Cell. Physiol.*, **136**, 398–410.

BELLONI, P.N. & TRESSLER, R.J. (1990). Microvascular endothelial cell heterogeneity: interactions with leukocytes and tumor cells. *Cancer Metastasis Rev.*, **8**, 353–389.

BELLONI, P.N., CARNEY, D.H. & NICOLSON, G.L. (1992). Organ-derived microvessel endothelial cells exhibit differential responsiveness to thrombin and other growth factors. *Microvasc. Res.*, **43**, 20–45.

BRUNSON, K.W. & NICOLSON, G.L. (1978). Selection and biologic properties of malignant variants of a murine lymphosarcoma. *J. Natl Cancer Inst.*, **61**, 1499–1503.

CAVANAUGH, P.G. & NICOLSON, G.L. (1989). Purification and some properties of a lung-derived growth factor that differentially stimulates the growth of tumor cells metastatic to the lung. *Cancer Res.*, **49**, 3928–3933.

CAVANAUGH, P.G. & NICOLSON, G.L. (1990). Purification and characterization of $M_r \sim 66,000$ lung-derived (paracrine) growth factor that preferentially stimulates the *in vitro* proliferation of lung-metastasizing tumor cells. *J. Cell. Biochem.*, **43**, 127–138.

CAVANAUGH, P.G. & NICOLSON, G.L. (1991). Lung-derived growth factor that stimulates the growth of lung-metastasizing tumor cells: identification as transferrin. *J. Cell. Biochem.*, **47**, 261–271.

CERRA, R.F. & NATHANSON, S.D. (1991). Chemotactic activity present in liver extracellular matrix. *Clin. Exp. Metastasis*, **9**, 39–49.

FIDLER, I.J. (1986). Rationale and methods for the use of nude mice to study the biology and therapy of human cancer metastasis. *Cancer Metastasis Rev.*, **5**, 29–49.

HORAK, E., DARLING, D.L. & TARIN, D. (1986). Analysis of organ specific effects on metastatic tumor formation *in vitro*. *J. Natl Cancer Inst.*, **76**, 913–922.

HUJANEN, E.S. & TERRANOVA, V.P. (1985). Migration of tumor cells to organ-derived chemoattractants. *Cancer Res.*, **45**, 3517–3521.

IRIMURA, T., TRESSLER, R.J. & NICOLSON, G.L. (1986). Sialoglycoproteins of murine RAW117 large cell lymphoma/lymphosarcoma sublines of various metastatic colonization properties. *Exp. Cell Res.*, **165**, 403–416.

JOSHI, S.S., TILDEN, P.A., JACKSON, J.D., SHARP, J.G. & BRUNSON, K.W. (1987). Cell surface properties associated with malignancy of metastatic lymphosarcoma cells. *Cancer Res.*, **47**, 3551–3557.

LABICHE, R.A., YOSHIDA, M., GALLICK, G.E., IRIMURA, T., ROBBERSON, D.L., KLOSTERGAARD, J. & NICOLSON, G.L. (1988). Gene expression and tumor cell escape from host effector mechanisms in murine large cell lymphoma. *J. Cell. Biochem.*, **36**, 393–403.

MCCARTHY, S.A., KUZU, I., GATTER, K.C. & BICKNELL, R. (1991). Heterogeneity of the endothelial cell and its role in organ preference of tumour metastasis. *Trends Pharmacol. Sci.*, **12**, 462–467.

MCGUIRE, E.J., MASCALI, J.J., GRADY, S.R. & NICOLSON, G.L. (1984). Involvement of cell-cell adhesion molecules in liver colonization by metastatic murine lymphoma/lymphosarcoma variants. *Clin. Exp. Metastasis*, **2**, 213–222.

MCMAHON, J.B., FARRELLY, J.G. & IYPE, T.P. (1982). Purification and properties of a rat liver protein that specifically inhibits the proliferation of nonmalignant epithelial cells from rat liver. *Proc. Natl Acad. Sci. USA*, **79**, 456–460.

MINER, K.M., WALTER, H. & NICOLSON, G.L. (1981). Subfractionation of malignant variants of metastatic murine lymphosarcoma cells by counter current distribution in two-polymer aqueous phases. *Biochemistry*, **20**, 6244–6250.

MINER, K.M. & NICOLSON, G.L. (1983). Differences in the sensitivities of murine metastatic lymphoma/lymphosarcoma variants to macrogphage-mediated cytolysis and/or cytostasis. *Cancer Res.*, **43**, 2063–2067.

NICOLSON, G.L., MASCALI, J.J. & MCGUIRE, E.J. (1982). Metastatic RAW117 lymphosarcoma as a model for malignant-normal cell interactions. *Oncodevelop. Biol. Med.*, **4**, 149–159.

NICOLSON, G.L. (1987). Differential growth properties of metastatic large cell lymphoma cells in target organ-conditioned medium. *Exp. Cell Res.*, **168**, 572–577.

NICOLSON, G.L. (1988*a*). Organ specificity of tumor metastasis: role of preferential adhesion, invasion and growth of malignant cells at specific secondary sites. *Cancer Metastasis Rev.*, **7**, 143–188.

NICOLSON, G.L. (1988*b*). Cancer metastasis: tumor cell and host organ properties important in metastasis to specific secondary sites. *Biochim. Biophys. Acta*, **948**, 175–224.

NICOLSON, G.L. (1989). Metastatic tumor cell interaction with endothelium, basement membrane and tissue. *Curr. Opin. Cell Biol.*, **1**, 1009–1019.

NICOLSON, G.L., BELLONI, P.N., TRESSLER, R.J., DULSKI, K., INOUE, T. & CAVANAUGH, P.G. (1989). Adhesive, invasive, and growth properties of selected metastatic variants of a murine large-cell lymphoma. *Invasion Metastasis*, **9**, 102–116.

NICOLSON, G.L., CAVANAUGH, P.G. & INOUE, T. (1992). Lung (paracrine) growth factors differentially stimulate the growth of lung-metastasizing tumor cells: identification of transferrin-like mitogens in lung tissue-conditioned medium. *J. Natl Cancer Inst.*, (in press).

PAGET, S., (1889). The distribution of secondary growths in cancer of the breast. *Lancet*, **i**, 571–573.

PALADE, G.E., SIMIONESCUE, M. & SIMIONESCUE, N. (1979). Structural aspects of the permeability of the microvascular endothelium. *Acta Physiol. Scand. Supp.*, **463**, 11–32.

PAULI, B.U., AUGUSTIN-VOSS, H.G., EL-SABBAN, M.E., JOHNSON, R.C. & HAMMER, D.A. (1990). Organ-preference of metastasis: the role of endothelial cell adhesion molecules. *Cancer Metastasis Rev.*, **9**, 175–189.

READING, C.L., BRUNSON, K.W., TORRIANNI, M. & NICOLSON, G.L. (1980*a*). Malignancies of metastatic murine lymphosarcoma cell lines and clones correlated with decreased cell surface display of RNA tumor virus envelope glycoprotein gp70. *Proc. Natl Acad. Sci. USA*, **77**, 5943–5947.

READING, C.L., BELLONI, P.N. & NICOLSON, G.L. (1980*b*). Selection and *in vivo* properties of lectin-attachment variants of malignant murine lymphosarcoma cell lines. *J. Natl Cancer Inst.*, **64**, 1241–1249.

READING, C.L., KRAEMER, P.M., MINER, K.M. & NICOLSON, G.L. (1983). *In vivo* and *in vitro* properties of malignant variants of RAW117 metastatic murine lymphoma/lymphosarcoma. *Clin. Exp. Metastasis*, **1**, 135–151.

REPESH, L.A. (1989). A new *in vitro* assay for quantitating tumor cell invasion. *Invasion Metastasis*, **9**, 192–208.

SUGARBAKER, E.V. (1981). Patterns of metastasis in human malignancies. *Cancer Biol. Rev.*, **2**, 235–278.

SZANIAWASKA, B., MAJEWSKI, S., MANINSKI, M.J., NOREMBERG, K., SWIERZ, M. & JANIK, P. (1985). Stimulatory and inhibitory activities of lung-conditioned medium on the growth of normal and neoplastic cells *in vitro*. *J. Natl Cancer Inst.*, **75**, 303–306.

TRESSLER, R.J., BELLONI, P.N. & NICOLSON, G.L. (1989). Correlation of inhibition of adhesion of large cell lymphoma and hepatic sinusoidal endothelial cells by RGD-containing peptide polymers with metastatic potential: role of integrin-dependent and -independent adhesion mechanisms. *Cancer Commun.*, **1**, 55–63.

TUCKER, R.F., SHIPLEY, G.D., MOSES, H.L. & HOLLEY, R.W. (1984). Growth inhibitor from BSC-1 cells closely related to platelet type b transforming growth factor. *Science*, **226**, 705–707.

VARANI, J. (1982). Chemotaxis of metastatic tumor cells. *Cancer Metastasis Rev.*, **1**, 17–28.

WEISS, L., ORR, F.W. & HONN, K.V. (1989). Interactions between cancer cells and the microvasculature: a rate-regulator for metastasis. *Clin. Exp. Metastasis*, **7**, 127–167.

YAMORI, T., IIDA, H., TSUKAGOSHI, S. & TSURUO, T. (1988). Growth stimulating activity of lung extract on lung-colonizing colon 26 clones and its partial characterization. *Clin. Exp. Metastasis*, **6**, 131–139.

YOSHIDA, M., GALLICK, G.E., IRIMURA, T. & NICOLSON, G.L. (1987). Modification of cell surface glycoproteins, macrophage, cytostasis, and blood-borne metastatic properties of the murine RAW117 large cell lymphoma by virus superinfection. *Cancer Res.*, **47**, 2558–2562.